# Supplementary material for: Metacognitive biases in anxiety-depression and compulsivity extend across perception and memory
Source: PLOS Ment Health. 2025 Mar 5;2(3):e0000259. doi: 10.1371/journal.pmen.0000259 (PMC12798496; doi:10.1371/journal.pmen.0000259)
Supplement: S1 File — (PDF) [file pmen.0000259.s001.pdf]

S1 File. Tutorial and task performance staircase titration.

Supplemental Figures

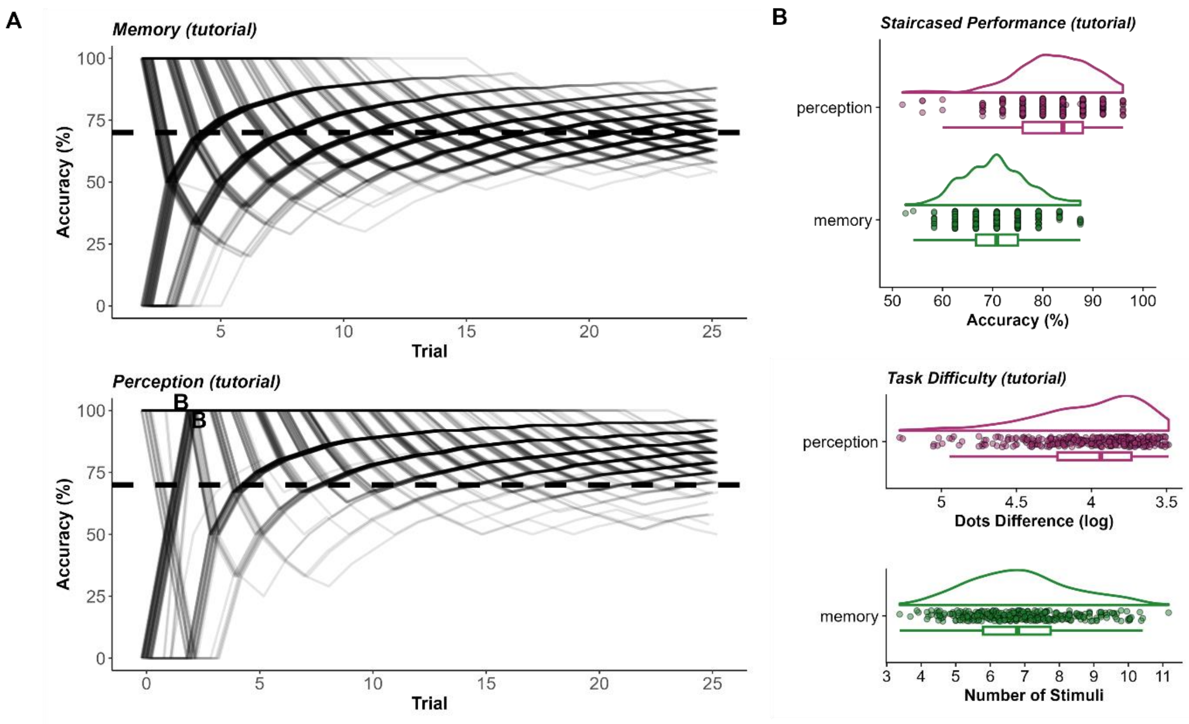

SFig 1. Tutorial (practice) performance titration via staircase procedure. We initiated the staircase procedure from the beginning of the practice trials. **(A) Mean accuracy over practice trials.** Accuracy was calculated as a sliding window including each trial over the accuracy of prior past trials. Each line represents a participant. At the end of the practice trials, the average performance was titrated close to the expected level in the memory task (M=70.59, SD=6.46), but not as much for the perception task (M=82.28, SD=7.50). Dotted lines represent 71% accuracy, where the staircase aimed to titrated performance to. **(B) Distributions of mean accuracy and task difficulty of the practice.** Participants on average finished the perceptual practice trials at a higher accuracy rate than the memory trials (t(325)=23.23, p<0.001, 95% CI=[10.70 12.68]). Task difficulty between tasks were correlated (r=-0.31, p<0.001, 95% CI=[-0.40 -0.21]).

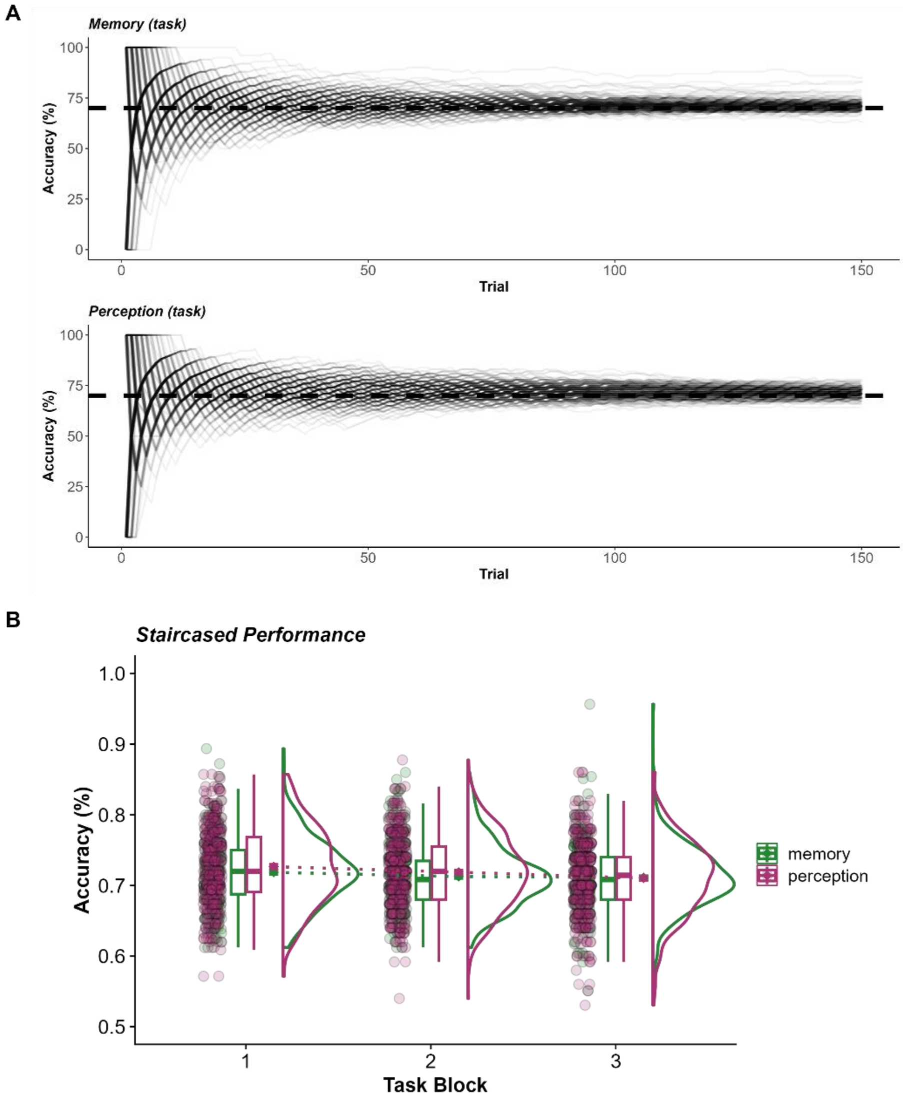

SFig 2. Task performance titration via staircase procedure. (A) Mean accuracy over task trials. Accuracy was calculated as a sliding window including each trial over the accuracy of prior past trials. Each line represents a participant. Performance in both tasks titrated to the expected level. Dotted lines represent 71% accuracy, where the staircase aimed to titrated performance to. **(B) Mean accuracy by task block.** We also examined accuracy within each of the three task blocks (50 trials each) for both tasks. Each circle indicates the accuracy of each participant per block.
