# Supplementary material for: Metacognitive biases in anxiety-depression and compulsivity extend across perception and memory
Source: PLOS Ment Health. 2025 Mar 5;2(3):e0000259. doi: 10.1371/journal.pmen.0000259 (PMC12798496; doi:10.1371/journal.pmen.0000259)
Supplement: S2 File — (PDF) [file pmen.0000259.s002.pdf]

S2 File. Harm versus Non-Harm stimuli effect on memory accuracy and confidence.

Supplemental Results

High compulsive individuals show blunted accuracy enhancement effect for harm versus non-harm stimuli

We established a link between memory confidence and compulsivity, and thus we further investigated this association. Previous clinical metamemory studies have shown a stronger underconfidence effect for the recollection of “unsafe” objects (i.e., stimuli perceived to be harmful and trigger OCD behaviours) versus neutral (i.e., OCD irrelevant) ones [1]. We hypothesized that high compulsive individuals may similarly show a reduction in memory confidence for items identified as “harmful” (harm) versus “not harmful” (non-harm). In our metamemory task design, we thus purposefully chose a selection of high and low harm rated images from a prior rating study [2] as stimuli (SFig 3).

We first tested if trial accuracy would vary if the correct answer was a harm stimulus (versus non-harm), controlling for the contribution of task difficulty, age, IQ and gender. We found that trials where harm stimuli were the target stimulus were more likely to be answered correctly ($\beta=0.11$, $SE=0.02$, $p<0.001$) and with higher confidence ($\beta=0.10$, $SE=0.009$, $p<0.001$) (SFig 4A), even when controlled for trial accuracy and difficulty. These findings replicate the boosting effect of “harmful” (affectively more salient) stimuli on memory, both in accuracy and confidence, even though the stimulus harmfulness was entirely task irrelevant.

We also hypothesized that high compulsive individuals would rate lower confidence on trials where harm items were the answer. We examined if confidence on each trial was linked to the stimulus category of the correct answer (controlling for trial accuracy, trial difficulty, age, IQ and gender). Contrary to expectations, we found no significant interaction effect of answer stimulus category with compulsivity on confidence ($\beta=0.0008$, $SE=0.009$, $p=0.93$) (SFig 4B), suggesting that there was no impact of harm versus non-harm items on their confidence. Instead, we found that individuals high in compulsivity showed a trending decreased trial accuracy enhancement effect for harm stimuli ($\beta=-0.05$, $SE=0.03$, $p=0.07$) (SFig 4B), where performance for harm and non-harm answer stimuli trials were more similar (SFig 3). This suggested that non-harmful stimuli were perceived as more similar to harmful stimuli in high compulsive participants.

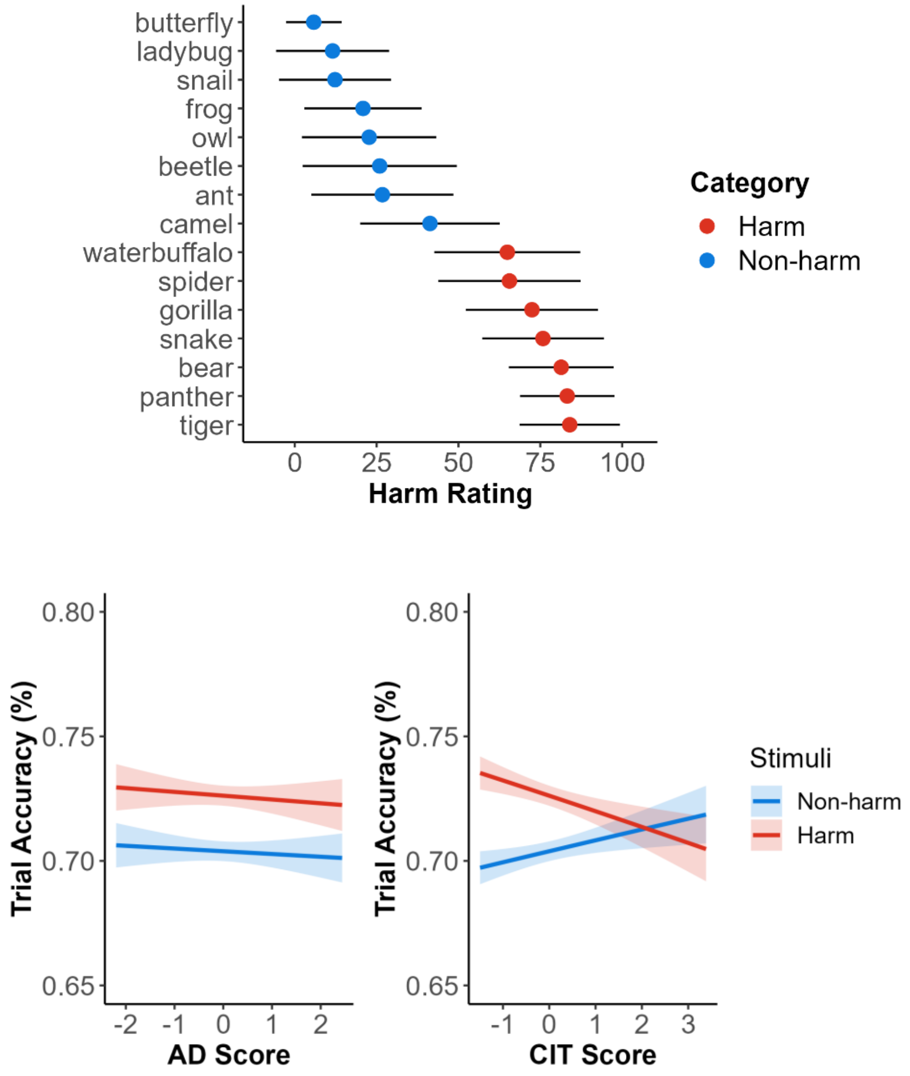

SFig 3. Harm levels of memory task stimuli and its relation to trial accuracy and dimension scores. In a prior affective rating study, we collected the harm ratings of the stimuli used in the memory task from N=80 participants [2]. Marker indicates the mean and standard deviation of the rating of each stimulus over two repeated presentations across participants. With the metamemory task, we assessed if the correct answer belonging to a harm versus non-harm category had an impact on the trial accuracy, and if it varied with dimension severity. Interaction effects show that high compulsive individuals do not show the accuracy enhancement effect for harm stimuli. AD: anxiety-depression, CIT: compulsivity.

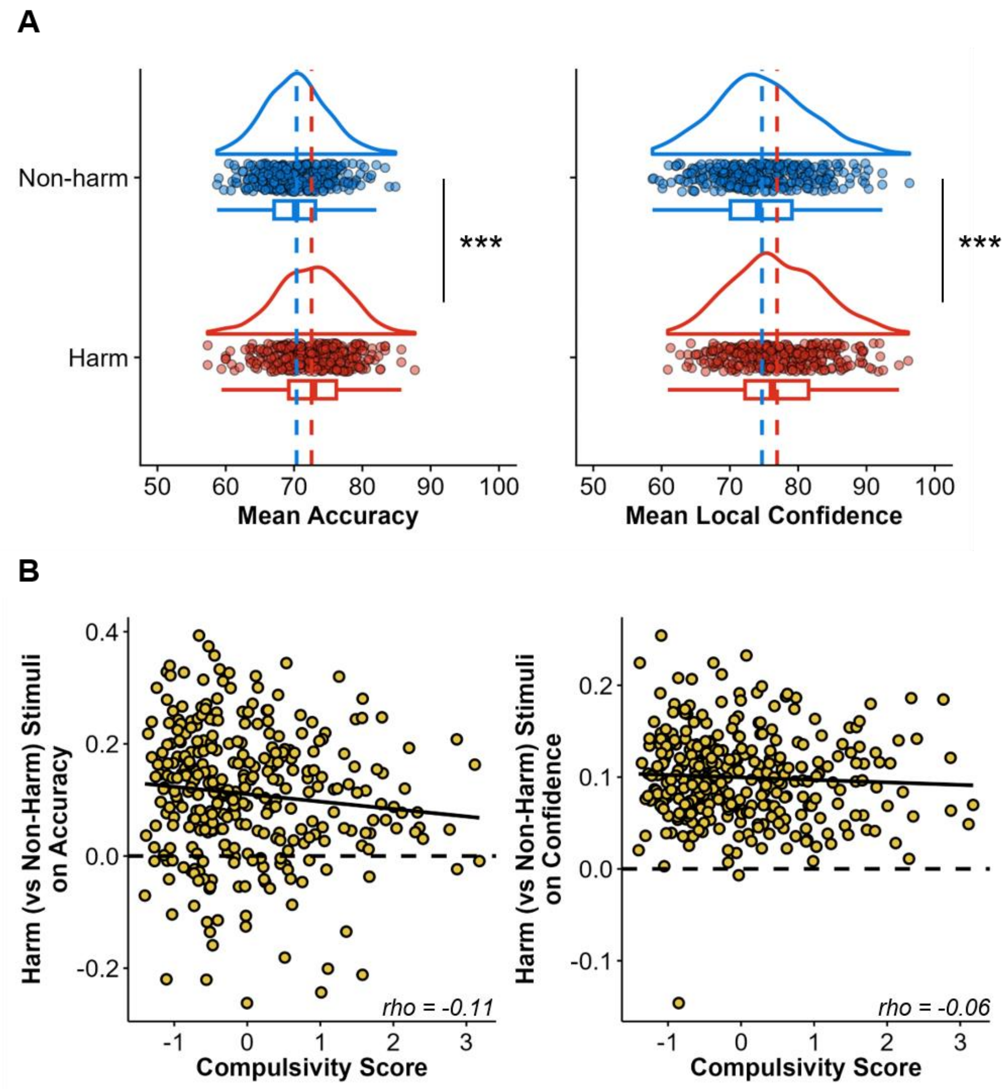

SFig 4. Performance and confidence distributions, and their relation to compulsivity scores, from the metaperceptual and metamemory task. (A) Mean accuracy and confidence of trials split by whether the correct answer was a harm or non-harm stimulus. Harm item trials were generally correct and had higher confidence ratings. Each circle represents each participant, the dotted lines represent the mean. ***$p < 0.001$. **(B) Correlation of compulsivity score with the stimuli category (harm versus non-harm) on accuracy or confidence**. For illustrative purposes, we obtained the accuracy or confidence residual controlled for anxiety-depression, social withdrawal, task difficulty, task accuracy (only for confidence), age, IQ and gender. The category*accuracy effect decreases as a function of increasing compulsivity scores, while category*confidence effect shows no significant relationship with compulsivity. Each circle represents each participant. Circles above the dotted lines indicate the boosting effect of harm (versus non-harm) stimuli on memory accuracy/confidence.

Supplemental Analyses

Mixed-effects modelling. To examine trial accuracy and confidence rating effects by stimuli category (Word Category; harm versus non-harm), we utilised a mixed-effects logistic regression model including number of stimuli presented (Task Difficulty), age, IQ and gender as main effect co-variates, with Word Category and Task Difficulty as random effect predictors. Task Difficulty, age and IQ were z-scored. The models were:

Trial Accuracy ~ Word Category + Task Difficulty + Age + IQ + Gender + (1 + Word Category + Task Difficulty | Subject)

Trial Confidence ~ Word Category + Trial Accuracy + Task Difficulty + Age + IQ + Gender + (1 + Word Category + Task Difficulty | Subject)

To examine if the effect of stimuli category on accuracy or confidence differed as a function of dimension scores, we interacted Word Category in the above model with the dimension scores. The models were:

Trial Accuracy ~ Word Category*(AD + CIT + SW) + Task Difficulty + Age + IQ + Gender + (1 + Word Category + Task Difficulty | Subject)

Trial Confidence ~ Word Category*(AD + CIT + SW) + Trial Accuracy + Task Difficulty + Age + IQ + Gender + (1 + Word Category + Task Difficulty | Subject)

Supplemental Discussion

Harm versus non-harm and compulsivity

Our metamemory task design allowed an exploratory analysis of the effect of harm versus non-harm answer stimuli on confidence. Prior work has implicated contextual effects of memory contents on its retrieval confidence in OCD [1], with a stronger underconfidence effect for remembering “unsafe” objects. We found that “harm” stimuli boosted memory performance in the group as a whole, but this effect was blunted in high compulsive individuals. It might be that individuals high in compulsivity identify all items as high in “harm”. Although we selected stimuli that were norm rated as “harmful” and “not harmful” [2], it might be more pertinent to use individual OCD-relevant stimuli for each participant in future work. While this is generally done in patients with OCD, it is more challenging in our dimensional approach with a general population where triggers are unknown and possibly less specific.

References

1. Tolin DF, Abramowitz JS, Brigidi BD, Amir N, Street GP, Foa EB. Memory and memory confidence in obsessive–compulsive disorder. Behaviour research and therapy. 2001;39: 913–927.

2. Seow TX, Hauser TU. What looks dangerous? Reliability of anxiety and harm ratings of animal and tool visual stimuli. Wellcome Open Res. 2024;9: 83.
