# Supplementary material for: Metacognitive biases in anxiety-depression and compulsivity extend across perception and memory
Source: PLOS Ment Health. 2025 Mar 5;2(3):e0000259. doi: 10.1371/journal.pmen.0000259 (PMC12798496; doi:10.1371/journal.pmen.0000259)
Supplement: S3 File — (PDF) [file pmen.0000259.s003.pdf]

S3 File. Meta-d' model parameter analyses.

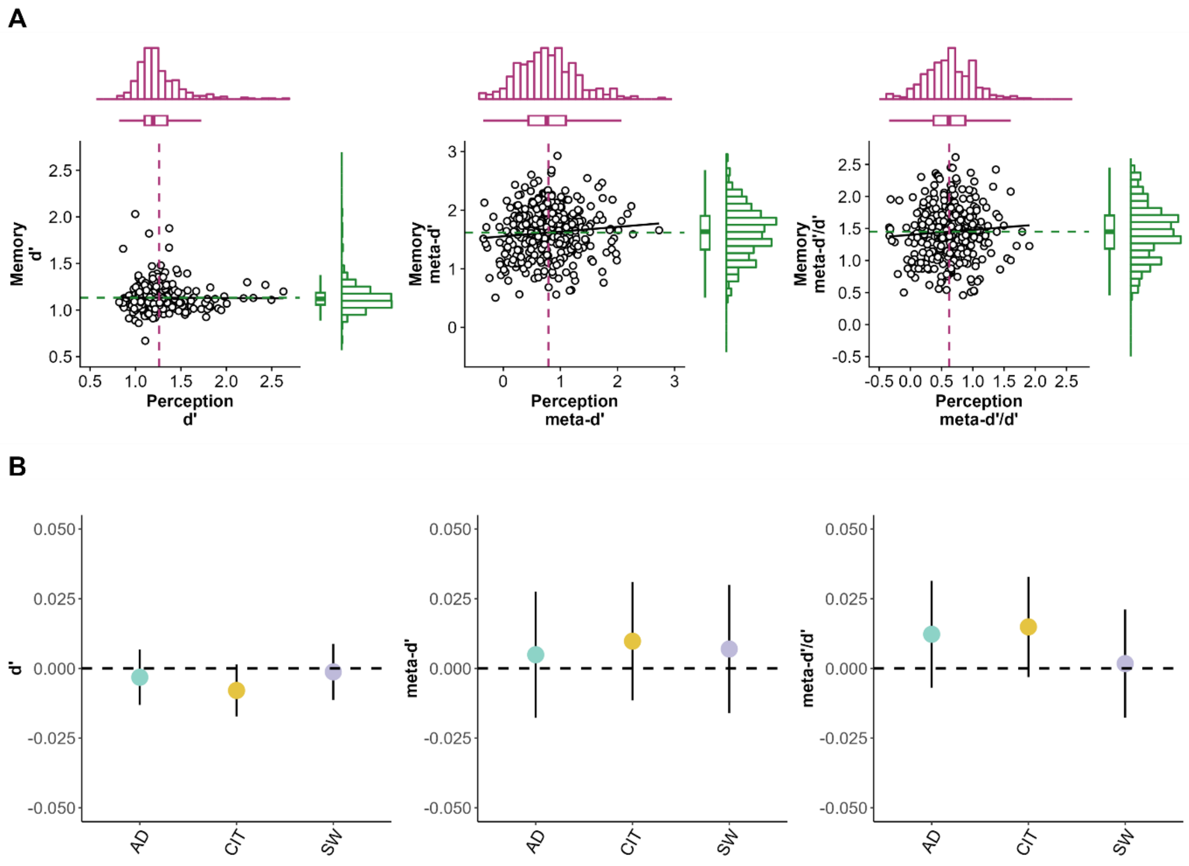

SFig 5. Individual meta-d' model parameter analyses and its relation to dimension scores. (A) Distributions of decision sensitivity (d'), metacognitive sensitivity (meta-d') and metacognitive efficiency (meta-d'/d'). We find lower d' (t(325)=-7.81, p<0.001, 95% CI=[-0.16 -0.10]), higher meta-d' (t(325)=23.41, p<0.001, 95% CI=[0.76 0.86]) and higher meta-d'/d' (t(325)=27.39, p<0.001, 95% CI=[0.77 0.89]) in the memory versus perception task. d' (r=-0.008, p=0.88, 95% CI=[-0.12 0.10]) and meta-d'/d' (r=0.07, p=0.19, 95% CI=[-0.04 0.18]) were not correlated between tasks, with only a trending positive correlation of meta-d' (r=0.10, p=0.08, 95% CI=[-0.01 0.20]). However, we note that d' patterns are more distributed, with several participants 3SD away from the mean d' in perception (N=6) and memory (N=5). **(B) Associations of decision sensitivity, metacognitive sensitivity and metacognitive efficiency with dimension scores.** We observed no significant relationships between any of the metrics with any dimension. The regression models were controlled for task domain, task order, age, IQ and gender.

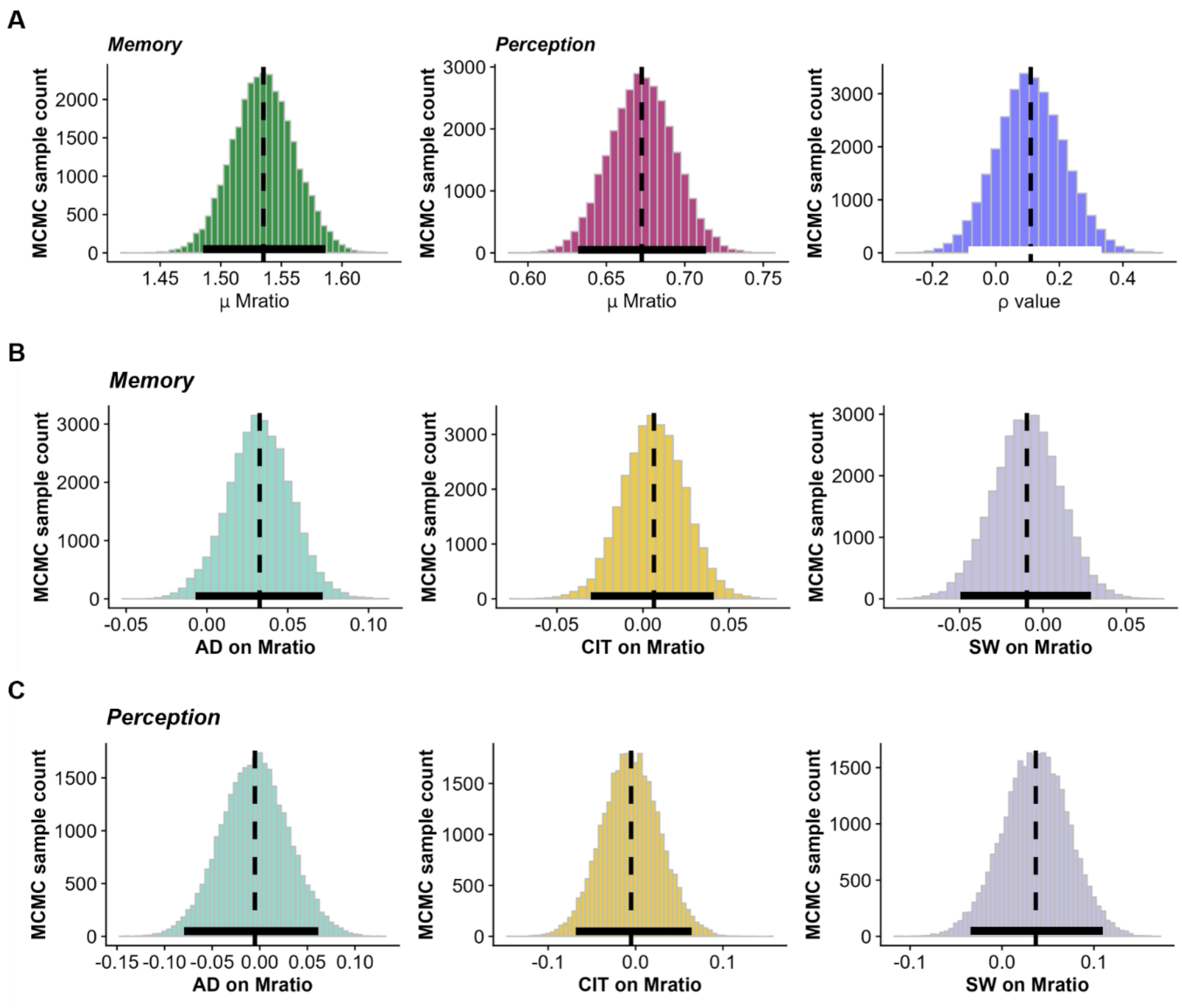

SFig 6. HMeta-d' model parameter analyses and their relation to dimension scores. (A) Markov chain Monte Carlo (MCMC) sample distributions for group level metacognitive efficiency (meta-d'/d') for the perception and memory tasks, and the correlation between the estimates from both tasks. As the reliability of single-participant meta-d'/d' estimation in SFig 6 can be low with small trial numbers, we ran the hierarchical model to get group level estimates. We found group level estimates were quite similar with the mean of the single-participant estimates for memory (mean meta-d/d'=1.54, 95% highest density interval (HDI)=[1.49 1.59]) and perception (mean meta-d/d'= 0.67, 95% HDI=[0.63 0.71]). We also found that the correlation between perception and memory group level meta-d'/d' estimates was positive (mean ρ=0.11, 95% HDI=[-0.10 0.31]). **(B) Associations of group level metacognitive efficiency of the memory task with dimension scores.** We conducted the regression of the dimension scores on metacognitive efficiency, controlled for age, gender and IQ, within the hierarchical model. We found that memory metacognitive efficiency was not linked to any of the dimensions (AD: mean=0.03, 95% HDI=[-0.007 0.07]; CIT: mean=0.006, 95% HDI=[-0.03 0.04]; SW: mean=-0.01, 95% HDI=[-0.05 0.03]). **(C) Associations of group level metacognitive efficiency of the perception task with dimension scores.** Similarly, we found that perceptual metacognitive efficiency was not linked to any of the dimensions (AD: mean=-0.005, 95% HDI=[-0.08 0.06]; CIT: mean=-0.005, 95% HDI=[-0.07 0.06]; SW: mean=-0.04, 95% HDI=[-0.03 0.11]).
