# Supplementary material for: Metacognitive biases in anxiety-depression and compulsivity extend across perception and memory
Source: PLOS Ment Health. 2025 Mar 5;2(3):e0000259. doi: 10.1371/journal.pmen.0000259 (PMC12798496; doi:10.1371/journal.pmen.0000259)
Supplement: S4 File — (PDF) [file pmen.0000259.s004.pdf]

S4 File. Distributions and correlations of questionnaire and dimensional scores.

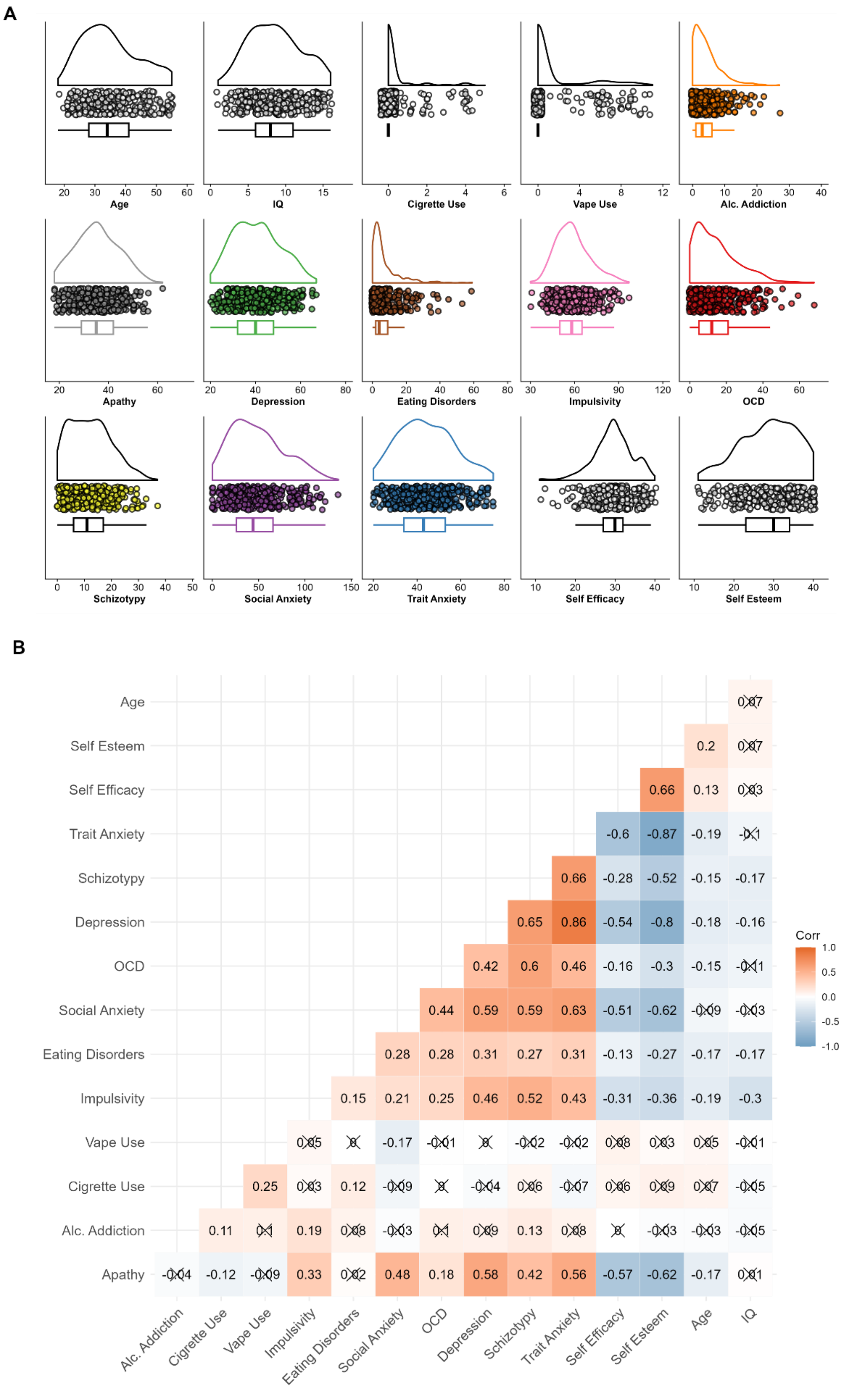

SFig 7. Age and questionnaire battery distributions and correlations. (A) Distributions of age and questionnaire scores. Each circle indicates an individual participant. **(B) Correlations between questionnaire scores.** Boxes that are crossed out indicate non-significant (p>0.05) correlations.

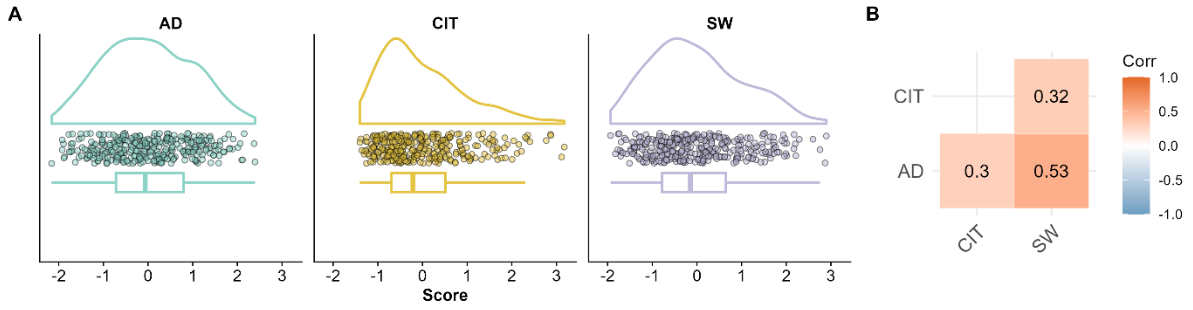

SFig 8. Transdiagnostic dimension score distributions and correlations. (A) Distributions of dimension scores. Each circle indicates an individual participant. **(B) Correlations between dimension scores.** All correlations are significant (p<0.001).

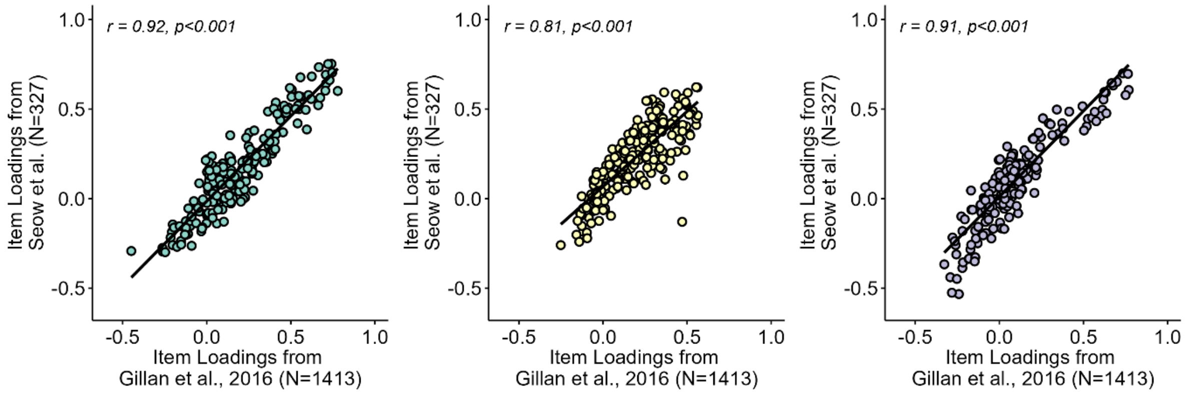

SFig 9. Correlations between item loadings for the three-factor structure from de novo factor analysis between the current sample (N=327) and Gillan et al. (2016) (N=1413). We find that even with a reduced sample size, we were able to replicate the same factor structure from the original analysis in Gillan et al. (2016). Each circle indicates an individual participant.
