# Supplementary material for: Metacognitive biases in anxiety-depression and compulsivity extend across perception and memory
Source: PLOS Ment Health. 2025 Mar 5;2(3):e0000259. doi: 10.1371/journal.pmen.0000259 (PMC12798496; doi:10.1371/journal.pmen.0000259)
Supplement: S5 File — (PDF) [file pmen.0000259.s005.pdf]

S5 File. Cross validation of regression models predicting dimension scores

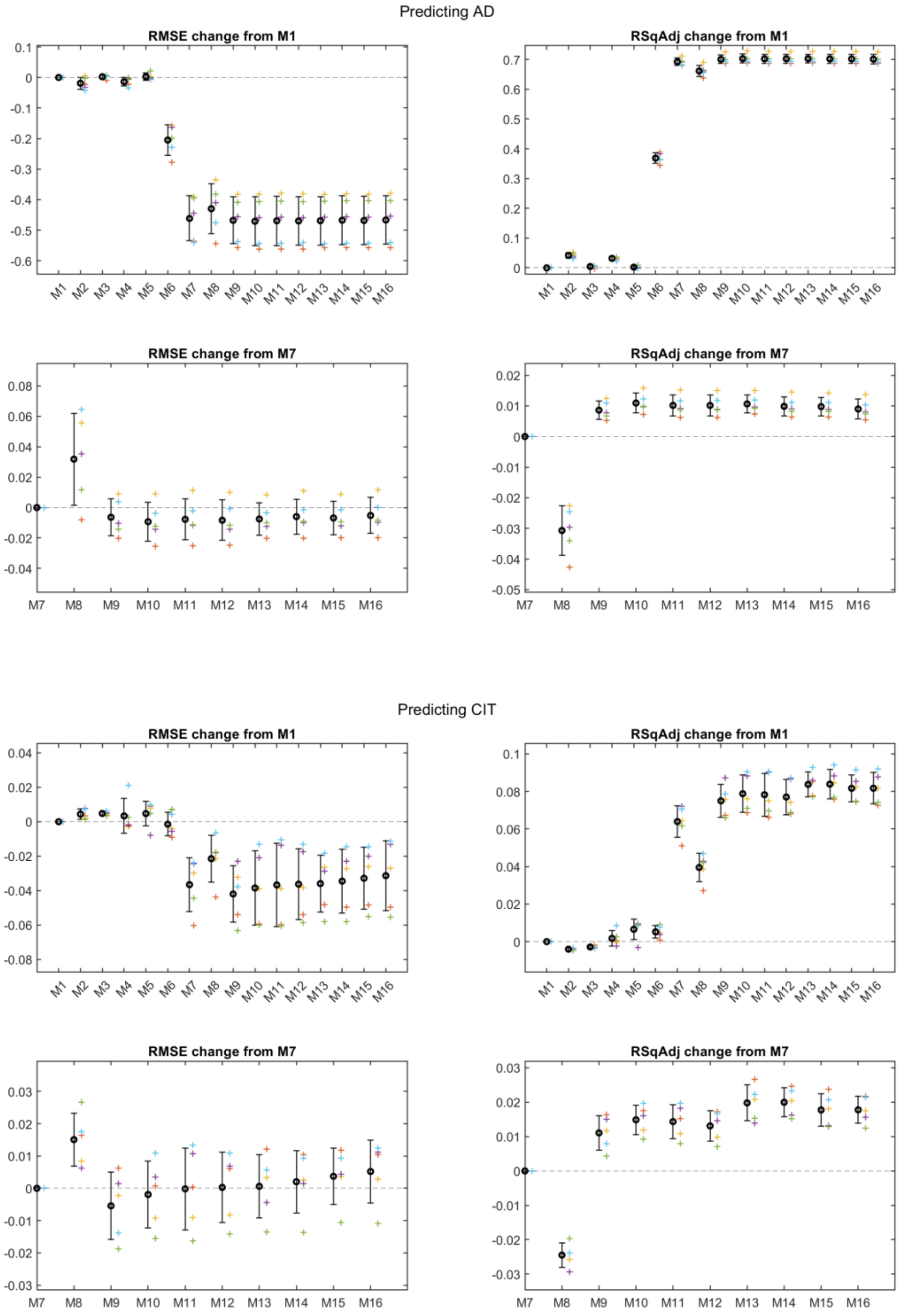

SFig 10. Cross validation of metacognitive models predicting anxiety-depression (AD) or compulsivity (CIT) severity. We examined 16 different models (see below) to predict dimension scores with a 5-fold cross validation procedure. Each coloured cross indicates model fit from a fold run, while the black marker indicates the mean and standard deviation of the model fits across the folds. We assessed both root mean square error (RMSE) as well as

the adjusted R^2 of the models. Model fits are depicted against a base model of demographics (age, gender and IQ; M1), as well as against a model of demographics with self-esteem (M7). For anxiety-depression, the winning model with the highest adjusted R^2 is M10. For compulsivity, the winning model with the highest adjusted R^2 is M13.

Models examined with cross validation:

- M1 = Dimension ~ Age + Gender + IQ
- M2 = Dimension ~ Age + Gender + IQ + Domain Metacognition
- M3 = Dimension ~ Age + Gender + IQ + Pre-task Metacognition
- M4 = Dimension ~ Age + Gender + IQ + Local Confidence
- M5 = Dimension ~ Age + Gender + IQ + Post-task Confidence
- M6 = Dimension ~ Age + Gender + IQ + Self-efficacy
- M7 = Dimension ~ Age + Gender + IQ + Self-esteem
- M8 = Dimension ~ Age + Gender + IQ + mean(Self-efficacy+Self-esteem)
- M9 = Dimension ~ Age + Gender + IQ + Self-efficacy + Self-esteem
- M10 = Dimension ~ Age + Gender + IQ + Self-efficacy + Self-esteem + Local Confidence
- M11 = Dimension ~ Age + Gender + IQ + Self-efficacy + Self-esteem + Local Confidence + Domain Metacognition
- M12 = Dimension ~ Age + Gender + IQ + Self-efficacy + Self-esteem + Local Confidence + Pre-task Metacognition
- M13 = Dimension ~ Age + Gender + IQ + Self-efficacy + Self-esteem + Local Confidence + Post-task Metacognition
- M14 = Dimension ~ Age + Gender + IQ + Self-efficacy + Self-esteem + Local Confidence + Post-task Metacognition + Domain Metacognition
- M15 = Age + Gender + IQ + Self-efficacy + Self-esteem + Local Confidence + Post-task Metacognition + Pre-task Metacognition
- M16 = Age + Gender + IQ + Self-efficacy + Self-esteem + Local Confidence + Post-task Metacognition + Pre-task Metacognition + Self-ability Metacognition

We chose the winning model as the one with the highest cross-validated (out-of-sample) adjusted R^2 and obtained the same results as the step-wise regression—the best model predicting anxiety-depression included self-esteem, self-efficacy and local confidence (R^2=0.75, RMSE=0.50, LLR=0.50), while the best model predicting compulsivity included self-esteem, self-efficacy, post-task metacognition and local confidence (R^2=0.23, RMSE=0.89, LLR=0.10). Note that local confidence, pre-task metacognition, post-task metacognition and self-ability metacognition regressors were interacted with task domain to control for cognitive domain effects.
