# Supplementary material for: Metacognitive biases in anxiety-depression and compulsivity extend across perception and memory
Source: PLOS Ment Health. 2025 Mar 5;2(3):e0000259. doi: 10.1371/journal.pmen.0000259 (PMC12798496; doi:10.1371/journal.pmen.0000259)
Supplement: S6 File — (PDF) [file pmen.0000259.s006.pdf]

S6 File. Task domain specificity of metacognition and behaviour.

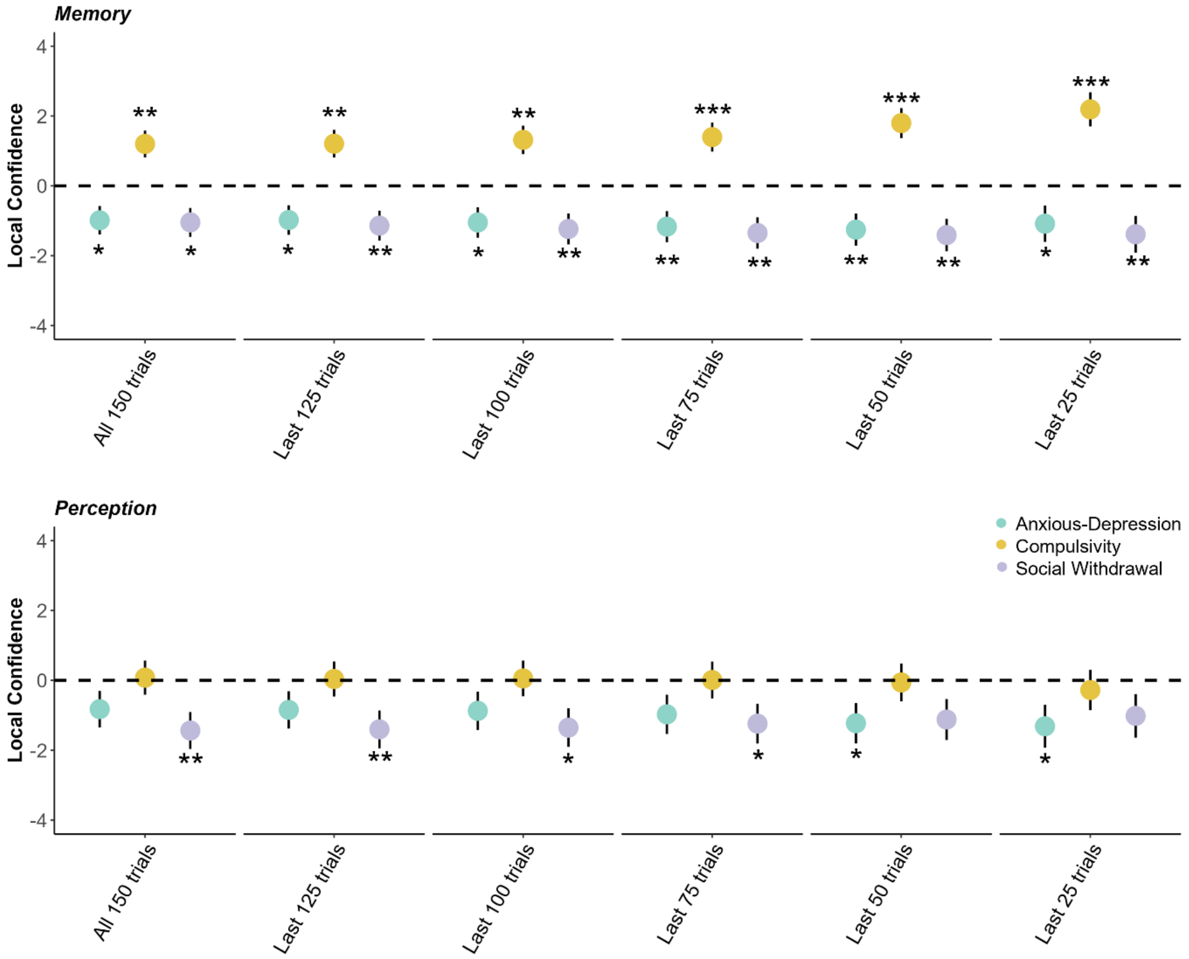

SFig 11. Regression of dimension scores on local confidence ratings for perception and memory over varying number of trials. The staircase procedure may have still required a burn-in for the beginning trials of the main task. We examined if associations of confidence with dimension scores would differ depending on the trials included in the analysis. We analysed the regression models separately for each task. All three dimension scores were included in the same model, which was controlled for task order, age, IQ and gender. We find similar patterns of results across each task even with varying trial numbers.

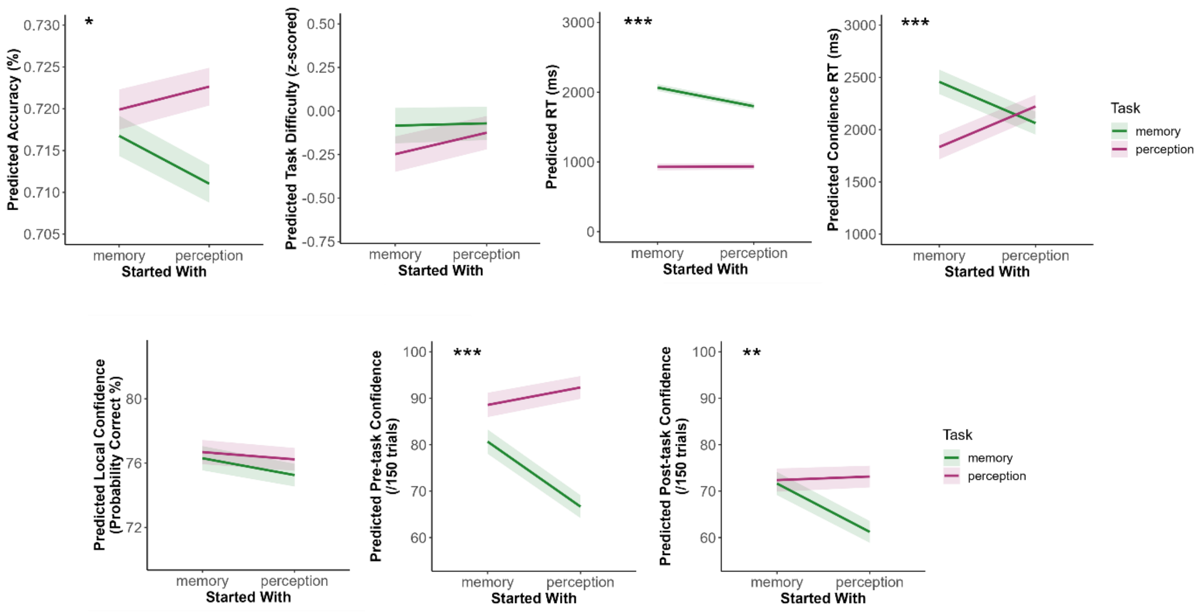

SFig 12. Task order effects. Participants were randomly assigned to complete either the metamemory or the metaperception task set first. Task order was taken as a control regressor in the main analyses, but showed some significant interaction effects with task domain in some analyses. Significance indicates significant interaction effect of task order with task domain on the dependent variable. *p<0.5, **p<0.01, ***p<0.001.
