# Supplementary material for: Metacognitive biases in anxiety-depression and compulsivity extend across perception and memory
Source: PLOS Ment Health. 2025 Mar 5;2(3):e0000259. doi: 10.1371/journal.pmen.0000259 (PMC12798496; doi:10.1371/journal.pmen.0000259)
Supplement: S7 File — (PDF) [file pmen.0000259.s007.pdf]

S7 File. Correlations between metacognitive measures across the hierarchy.

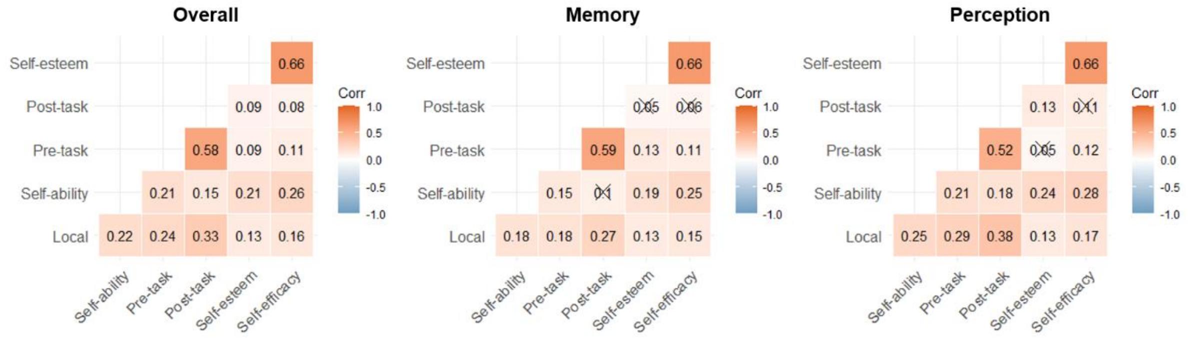

SFig 13. Correlations between metacognitive metrics. Pearson's correlations between the various metacognitive metrics obtained from the task—local (trial-by-trial task confidence), pre-task metacognition, post-task metacognition, self-ability metacognition as well as questionnaire scores of self-esteem and self-efficacy.
