# Supplementary material for: Metacognitive biases in anxiety-depression and compulsivity extend across perception and memory
Source: PLOS Ment Health. 2025 Mar 5;2(3):e0000259. doi: 10.1371/journal.pmen.0000259 (PMC12798496; doi:10.1371/journal.pmen.0000259)
Supplement: Attachment — Submitted filename: Response to Reviewers.pdf [file pmen.0000259.s008.pdf]

Response to Reviewers

Editor Comments to the Author

Q.0.1: Based upon the reviews, and my own consideration of the paper, we feel that you work has merit but does not fully meet PLOS Mental Health's publication criteria as it currently stands. Therefore, we invite you to submit a revised version of the manuscript that addresses the points raised during the review process.

As you will note upon consulting the comments, experts identified strengths in your work, expressed an overall positive evaluation of it, and acknowledged the importance of the topic. Whereas Reviewer#1 only has minor points to address, Reviewer#2 identified major issues, some quite serious, that require your attention. You will find some of these points below for your consultation. However, please note that the integrality of the Reviewer #2's review is attached to this message as a pdf document (Review_2024-09-09). Overall, Reviewer#2 identifies weaknesses and ambiguities in the argumentative flow of the manuscript and, among other things, requests that the appearance of new concepts all along the text be contextualised in a more rigorous manner. The reviewer also highlights a lack of justification for the statistical tools used so as a lack of details in the description of the analyses and the reporting of the results, which unfortunately blurs the relevance of the researchers' analytical strategy and casts doubt on the coherence of the observed effects.

R.0.1: We thank the editor for their positive impression of the manuscript and the opportunity to submit our revised paper. We acknowledge the comments raised from both reviewers, and acknowledge the lack of clarity on the concepts and analyses within the text. We have taken care to address each of the listed points below, including those of the attached PDF document. To more accurately characterise one of our mental health factors, we have also renamed 'anxious-depression' to anxiety-depression' throughout the paper.

We believe that the current paper is much clearer and stronger after the advised revision, and hope that we have adequately addressed all concerns.

Q.0.2: On my part, I must also bring to your attention that the organization of your manuscript is not in line with the PLOS Mental Health submission guidelines, that you can find here: https://journals.plos.org/mentalhealth/s/submission-guidelines. I therefore encourage you to modify the manuscript accordingly.

R.0.2: We have moved the Methods section prior to the Results, as well as reorganised the Supplementary Materials to have them fully in accordance to the submission guidelines. As such, we also believe that the concepts are detailed more clearly before the results.

Review Comments to the Author

Reviewer #1:
Q.1.1: The methodology used can provide valuable insights with regard to the research questions. However, the paper lacks overall clarity and precision, especially with regard to its objectives and methodology description. These elements must be clarified to properly assess the appropriateness of the conclusions. The analyses are not sufficiently detailed, and it is unclear which specific analysis was used to address each research question.

Further comments (from PDF): This study evaluates the associations between 1) different metacognitive monitoring measures, more or less global according to a hierarchy of metacognitive processes, and 2) measures of "anxio-depression" and "compulsivity" across

perception and memory. The methodology involves two two-alternative decision-making tasks associated with trial-by-trial confidence ratings (i.e., a metamemory task and a metaperception task), along with a set of questionnaires measuring psychopathology, self-esteem, self-efficacy, pre- and post-task self-performance estimates, and self-ability estimates, in a sample of N=327 online participants from general population. The topic of this study is important, and the paradigms used can provide valuable insights in this regard.

However, the paper lacks overall clarity and fails to explain the study objectives clearly. It also lacks detailed methodology descriptions. It seems that the methodology does not allow the study to meet its objective of "disentangling the conflicting findings relating metacognitive biases to compulsivity and OCD," as no sample of patients diagnosed with OCD was included. One possibility is that the notion of "compulsivity," as used in this article—encompassing a range of "compulsive-type symptoms" such as addictions and eating disorders—may have little relation to OCD in terms of metacognition.

Additionally, the paper lacks overall clarity and precision, particularly regarding the notions used, the study objectives, and the methodology. It is difficult to determine which method (analysis) and which result addresses each research question. The terminology is often confusing (e.g., the mean of trial-by-trial confidence ratings is sometimes referred to as "metacognitive bias," "confidence," or "local task confidence").

Furthermore, I have doubts about the relevance of some analyses and whether they support the conclusions the authors draw.

R.1.1: We thank the reviewer for their insightful comments. We agree that the paper overall required better clarity, especially in terms of the (i) concepts listed, (ii) the study objectives and (iii) the methodology. We have covered all these areas in detail further below, and hope they are now clear.

We also address how 'compulsivity' relates to OCD. Prior work has demonstrated that the same compulsivity construct we define here captures known OCD cognitive deficits such as poor goal-directed learning (Gillan et al., 2016; eLife), and may have greater biological validity than OCD diagnosis itself (Gillan et al., 2019; JAMA Psychiatry). Interestingly, using data from a clinical sample also produces the same three transdiagnostic factors as general population samples (Fox et al., 2024; OSF). These points speak highly of the clinical validity of the compulsivity construct and that this also likely holds in a clinical context. We now highlight this point in the introduction:

> *"Notably, clinical samples also produce these three transdiagnostic dimensions [19]. In particular, the 'compulsivity' construct not only includes compulsive behaviours characteristic of OCD, but also encompasses other compulsive-type symptoms such as addiction and eating disorders [18]. These features have been shown to have greater biological validity in relation to known OCD cognitive deficits than diagnosis itself [20]."*

Q.1.2: (Further comments from attached PDF) Introduction: The introduction is difficult to follow. The authors propose three possible explanations for the issue of divergent results in the literature. At the end of the introduction, the study's results are briefly presented, but they only address two of these hypotheses, as no sample of patients suffering from OCD is included.

R.1.2: We intended to transparently state all possible explananda for the divergent results in the literature, although we acknowledge that we are unable to directly test the one hypothesis of transdiagnostic versus case-control methods due to the lack of a clinical OCD sample.

Using a transdiagnostic methodology, our study addresses this hypothesis only indirectly by suggesting that the overshadowing effect is possible, in the absence of a big patient cohort. We have highlighted this in the discussion, but we now also mention this explicitly in the introduction:

> *"We focused on using a transdiagnostic individual differences approach with a general population (but not explicit patient) sample, and tested metacognition across the cognitive domains of perception and memory, and across a hierarchy of metacognitive measures."*

Q.1.3: Moreover, and this is likely a drawback of presenting results before the methods section, the results presented in the introduction are hard to understand. Most of the specific terms used are not defined beforehand.

R.1.3: We agree that the arrangement of results before methods made the text more difficult to follow, and have now switched this, taking care to note that all the specific terms are defined.

Q.1.4: Line 46: Lowered confidence in decisions/actions does not imply "metacognitive bias." The latter should refer, in this case, according to the authors' definition of metacognitive bias, to situations where lowered confidence diverges from reality.

R.1.4: We apologise for the confusion about the definition of the term here. We broadly defined "metacognitive bias" as "self-evaluations diverge from reality" in the introduction. Deviations from reality can be either absolute (as when a probabilistic judgment of confidence diverges from % correct), or relative, as when an experimental or individual difference factor introduces variance in confidence that is not manifested in performance. Our paradigm is an example of the latter, as performance is staircased to the same level across individuals. Thus, individuals that rate lower confidence in this task relative to the mean (of the sample) can be said to exhibit metacognitive bias in relative (not necessarily absolute) terms, given that they report a self-evaluation that is "less than" the rest, despite equal levels of performance.

This terminology adheres to that which is commonly used and accepted in the field for at least a decade now. Concretely, mean confidence level is a well-known measure of metacognitive bias (separable from metacognitive sensitivity, how confidence tracks the accuracy of responses) in metacognitive research in cognitive neuroscience (see Fleming & Lau, Frontiers in Human Neuroscience, 2014). We appreciate that this was not explicitly detailed in the original paper, leading to the ambiguity. We have now added this to the revised introduction:

> *"Empirically, this entails examining if there are biases in the summary statistics (e.g., mean) of trial-by-trial confidence ratings within a cognitive task where performance is equated across participants through a staircase procedure [3]."*

Q.1.5: Line 50: Specify that these studies have also examined confidence in areas other than memory, such as in perceptual decision tasks. Otherwise, the explanation provided in L58 is not clearly understood.

R.1.5: We have now rectified the sentence to:

> *"However, recent studies have observed more differentiated effects when adopting a transdiagnostic approach with general population samples in metacognitive research using perceptual or reinforcement learning decision tasks [17]."*

Q.1.6: Line 55: Clarify: "...transdiagnostic compulsivity is associated with overconfidence in studies using memory paradigms, whereas a diagnosis of OCD is associated with underconfidence in studies using tasks that engage other cognitive domains."

R.1.6: We have modified this line to:

> *"Taken together, these findings suggest a striking and hitherto unexplained dissociation in which a diagnosis of OCD is linked to underconfidence using memory paradigms, whereas transdiagnostic compulsivity is linked to overconfidence in other cognitive tasks beyond memory."*

Q.1.7: Line 63: Should read: "the metacognitive domain-generality in perception and episodic/working memory in compulsivity."

R.1.7: We have added the word "metacognitive" to the line:

> *"… the metacognitive domain-generality of perception and episodic/working memory in compulsivity is untested and key to the current question."*

Q.1.8: Line 82: A possibility is that confidence varies across domains (e.g., memory vs. perceptual task) among OCD patients but not among "compulsive" individuals. This study does not address that.

R.1.8: We thank the reviewer for raising this point. Indeed, this study does not address that, given the lack of an OCD sample; we intended to target the more basic and obvious difference of the differing domains in metacognition here. However, we note this possibility in the discussion:

> *"It is thus also possible that there are specific features in patients that make them categorically distinct from "non-patients" [63] and lead to metacognition varying across domains differently between these two cohorts, or that other forms of co-morbid psychopathology could explain this lowered confidence."*

Q.1.9: Lines 84 to 89: Many terms are not defined in the introduction, and their meaning can only truly be understood after reading the entire article: "global self-esteem," "global task confidence," "self-efficacy," "local task confidence."

R.1.9: We apologise for this confusion. We now define these terms in the introduction:

> *"We quantified six measures spanning local to global metacognition—from local (trial-by-trial) task confidence ratings, to global measures of pre- and post-task evaluations of performance, to even more global estimates of cognitive domain ability, to the highest-level global metacognitive measures of self-esteem and self-efficacy."*

Q.1.10: Results: Line 112: "Local task confidence is closely linked" is not a clear expression.

R.1.10: The sub-heading has been replaced to "Local task confidence is closely correlated across perception and memory".

Q.1.11: Line 157: "Mean trial-by-trial confidence ratings" is not a measure of metacognitive bias.

R.1.11: Please see our detailed response in **R.1.4**, where we describe how this terminology is consistent with the accepted usage in the literature. The mean of trial-by-trial confidence ratings is considered a (relative) measure of "metacognitive bias".

Q.1.12: Line 170: The term "link" is not appropriate for designating statistical associations.

R.1.12: We have replaced the term "link" with "association".

Q.1.13: Line 174: Depression, social anxiety, trait anxiety, and schizotypy are not mood dysregulation symptoms.

R.1.13: We apologise for the inaccurate term and recognise that disruptive mood dysregulation disorder refers to a different mental health condition. We meant to indicate that these symptoms are present in mental health disorders that are often characterised by mood alterations, particularly of low mood. We have now reworded the sentence to reflect the original intention better:

> *"…we observed that several questionnaires, particularly subclinical traits that are central to disorders with low mood (e.g., apathy, depression, social anxiety, trait anxiety), were linked to lower confidence levels (β<-0.11, SE<0.04, p<0.005) across perception and memory (Fig 3A)."*

Q.1.14: Line 177: At this point, the argument would be more understandable if we had a clearer idea of what the notions of anxious-repression, compulsivity, and social withdrawal refer to (beyond the fact that they are factors).

R.1.14: We agree this was unclear previously. We now add a short explanation in the introduction:

> *"These studies, including our own, utilised an established set of latent factors of psychopathology encompassing 'anxiety-depression', 'compulsivity and intrusive thoughts' (hereafter: 'compulsivity'), and 'social withdrawal' dimensions, derived using factor analysis [18]."*

Additionally, we have moved methods to come before the results, where the section on **Transdiagnostic dimensions** describes their quantification. We also added a sentence in the methods that introduces what each dimension encompasses:

> *"The prior study reported that the highest average item loadings came from the trait anxiety, apathy and depression questionnaires for the anxiety-depression factor, from the eating disorders, addiction and OCD questionnaires for the compulsivity factor, and from the social anxiety questionnaire for the social withdrawal factor."*

Q.1.15: Line 193: The rationale for showing results concerning social withdrawal is not discussed. It is not part of the study's objective.

R.1.15: While we agree that results of the social withdrawal dimension are not part of the study objective, prior studies using the transdiagnostic approach have all reported observations for

all three dimensions (see Rouault et al., 2018; Benwell et al., 2022; Hoven et al., 2022, etc.). Social withdrawal has repeatedly not shown any associations with metacognitive bias, and thus the lack of effect is also an expected result. We thus likewise opted show all three dimensions in this manuscript for full transparency of the results and completeness, particularly since we observed an unexpected association of higher social withdrawal scores predicting lower confidence levels. We previously discussed this in the supplementary file, under "Local confidence and social withdrawal". We have now moved this discussion to the main text:

> *"We unexpectedly observed that social withdrawal exhibited similar associations with metacognition as those found for anxiety-depression, which was never seen before in prior work [21,22,26]. Although we found that social anxiety questionnaire score distributions in the current sample were similar to those of our prior study [26], we also found that correlations between the anxiety-depression and social withdrawal factors were higher than expected (r=0.53 versus r=0.43 [26] or r=0.33 [22]). Indeed, when social withdrawal was not included in the regression models, our anxiety-depression effects became much stronger (e.g., β=-0.12 to β=-0.20 when predicting local confidence). As social withdrawal has not shown significant relations with metacognition in any of the prior studies using the full three-factor transdiagnostic approach [21,22,26] we refrain from interpreting these effects further and focus on anxiety-depression and compulsivity."*

Q.1.16: Line 198: Task accuracy is set by design (staircase procedure). Is there enough between-subject variation in task accuracy to examine its association with symptom dimensions?

R.1.16: Indeed, task accuracy is staircased, and thus there should not be large between-subject variation in task accuracy. We examined the association of accuracy with symptom dimensions nonetheless as a replicative sanity check. We noted this in the main text:

> *"Finally, to ensure that these effects were not driven by task performance, we established that task accuracy (ps>0.44) and difficulty (ps>0.16) did not show significant relationships with symptom dimensions."*

Prior studies (e.g., Rouault et al., 2018) likewise have shown no effects, and should there be a signification association, it would imply that the staircase did not work as intended.

Q.1.17: Line 200: This result should indeed be moderated by the fact that there is a trend for the Compulsivity X task domain interaction.

R.1.17: We have added this moderation:

> *"Overall, our findings thus suggest that relationships between mental health dimensions and local task confidence are relatively similar across task domains (albeit a stronger association with memory than perception), supporting a domain-general relationship between metacognition and psychopathology."*

Q.1.18: Line 218: Not "controlling for" but "adjusting for," same for Line 237.

R.1.18: We have replaced all instances of "controlling" to adjusting":

“However, recent studies with OCD patients have found lowered confidence in comparison to healthy controls despite adjusting for anxiety and depression scores [61,62].”

“Mean confidence ratings were highly positively correlated across tasks (r=0.65, p<0.001, 95% CI= [0.59 0.71]), and remained significantly correlated even after adjusting for task difficulty (r=0.48, p<0.001, 95% CI= [0.39 0.56]), supporting a domain-general pattern of confidence.”

“We first assessed the association between mean confidence level and each self-report questionnaire score we collected, adjusting for the influence of task domain, task completion order, age, IQ and gender.”

“We then tested if these global metacognitive estimates differed as a function of dimension scores, adjusting for task domain and completion order (if relevant), age, IQ and gender.”

“For illustrative purposes, we obtained the confidence residuals for each participant after adjusting for anxiety-depression/compulsivity (anxiety-depression if compulsivity and confidence was to be examined, and vice versa), social withdrawal, task order, age, IQ and gender, separately for each task.”

Q.1.19: Lines 228-237: There is a missing explanation (potentially with an illustration) of this "metacognitive hierarchy." It is also not clearly indicated how the various presented measures are positioned within this hierarchy.

R.1.19: We agree this could be clearer. We now add a new inset D into Fig 1 that illustrates the metacognitive hierarchy with all six metacognitive measures positioned within it.

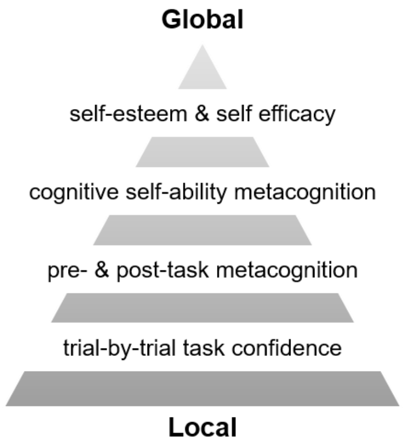

***Fig 1. (D) Metacognitive measures.** We quantified six different measures spanning local to global estimates across the metacognitive hierarchy. (Mean of) Trial-by-trial confidence ratings were considered the most “local” as they were reflections of a single decisions, while pre- and post-task metacognition were more “global” entailing reflections about an entire task. Even more global cognitive self-ability metacognition captured the evaluation of an entire cognitive domain, whilst the most global levels of self-esteem or self-efficacy measures reflect a combination of cognitive self-evaluations with other trait influences.*

Q.1.20: Lines 239-251: What analyses are these results derived from? They are not mentioned or detailed in the methods.

R.1.20: We apologise for the oversight in excluding the methods for these analyses. They follow the same model as mean local confidence; the general linear modelling section in the Methods now details all of this:

> *"We also assessed if global levels of metacognition (Global Metacognition) were associated with dimension (Dimension) scores. These included pre-task metacognition, post-task metacognition, self-performance estimates, self-esteem and self-efficacy scores. We utilised the same linear model as above, replacing the dependent variable with each global metacognition construct of interest. For pre-task metacognition, post-task metacognition and self-performance estimates, we estimated a score each for memory and perception. Thus, the models were:*
>
> *Global Metacognition ~ (AD + CIT + SW + Task Order + Age + Gender + IQ)*Task Domain*
>
> *For self-esteem and self-efficacy, as there was only one score per participant (no domain dependency), the models were:*
>
> *Global Metacognition ~ (AD + CIT + SW + Task Order + Age + Gender + IQ)*
>
> *The main effect of AD/CIT/SW (Dimension) on Global Metacognition was interpreted as a general metacognitive effect of psychopathology."*

Q.1.21: Line 276: "Differing local and global cumulative metacognitive contributions..." is unclear.

R.1.21: We have rewritten this sub-heading to "Local and global metacognitive contributions to anxious-depression and compulsivity differ".

Q.1.22: Line 285: The authors' conclusions focus on the relative contributions of the predictors in the models. This is actually the result of a qualitative interpretation of the magnitude of the regression coefficients. The analysis method used here does not allow for statistical comparison of the regression coefficients with each other.

R.1.22: We utilised the stepwise regression approach, which includes regressors in a stepwise manner based on model fit. This approach includes regressors into the model by means of measuring a better model fit—in other words, the order in which the regressors were added were determined by a statistical test of better model fit (AIC). However, we do agree that this is not explicit in the current text. We have now included the change in AIC when comparing each of the regressors additive effect onto predicting confidence:

> *"For anxiety-depression, low self-esteem (β=-0.76, SE=0.03, p<0.001; ΔAIC=-853.62) was the biggest contributor, followed by low self-efficacy (β=-0.11, SE=0.03, p<0.001; ΔAIC=-20.53), and low local task confidence (β=-0.06, SE=0.02, p=0.003; ΔAIC= -6.98). For compulsivity, the most impactful predictor was again low self-esteem (β=-0.34, SE=0.04, p<0.001; ΔAIC=-49.83), then high self-efficacy (β=0.13, SE=0.04, p=0.002; ΔAIC=-8.14), low post-task metacognition (β=-0.07, SE=0.04, p=0.04; ΔAIC=-0.48) and high local task confidence (β=0.06, SE=0.04, p=0.12; ΔAIC=-0.43)."*

Q.1.23: Line 343: Using a "dimensional approach" does not guarantee that comorbid anxiety/depression symptoms are accounted for. This is ensured by including anxiety/depression symptoms in the analysis.

R.1.23: We thank the reviewer for raising this point of discussion. We do indeed include comorbid anxiety/depression symptoms in our regression analyses—all models that examine the dimension score impact on metacognition includes all three scores (anxiety-depression, compulsivity and social withdrawal).

Moreover, we would like to highlight that the current dimensional approach accounts for comorbid symptoms over and above including them in the regression model, that traditional methods are not able to do. By using the factor analysis, we extract latent dimensions that best captures the variance of the questionnaire data. This is to especially account for the fact that many questionnaires measure co-varying symptoms. For instance, the OCI-R (measures obsessive-compulsive symptoms) also has question items reflecting anxiety/depression-like symptoms (e.g. "I find it difficult to control my own thoughts."), leading to high collinearity in questionnaire scores (OCI-R & STAI-Y2/SDS: r=0.42-0.46). However, when factor analysis is applied to the questionnaires item scores, it produces the three dimensions of anxiety-depression, compulsivity and social withdrawal that each capture their named psychopathology with less covariance with others. In this case, anxiety-depression and compulsivity now correlates at r=0.30.

Q.1.24: Line 345: "It is thus also possible that there are specific features in patients that make them categorically distinct from 'non-patients.'" See the results of the following article, which suggest this regarding metacognition: "Baptista, A., Maheu, M., Mallet, L., & N'diaye, K. (2021). Joint contributions of metacognition and self-beliefs to uncertainty-guided checking behavior. Scientific Reports, 11(1), 19017."

R.1.24: We thank the reviewer for bringing this article to our attention, which we now cite as ref 63:

> *"It is thus also possible that there are specific features in patients that make them categorically distinct from "non-patients" [63] and lead to metacognition varying across domains differently between these two cohorts, or that other forms of co-morbid psychopathology could explain this lowered confidence."*

Q.1.25: Line 349: More details about the notion of "compulsivity" should be given in the introduction.

R.1.25: We have now added in the Introduction:

> *"In particular, the 'compulsivity' construct not only includes compulsive behaviours characteristic of OCD, but also encompasses other compulsive-type symptoms such as addiction and eating disorders [18]."*

Q.1.26: Line 352: "Not particularly strong": specify what this means in terms of effect size.

R.1.26: We now add in a comparison of the effect size between compulsivity and AD:

> *"We note that the association between high confidence and compulsivity was not particularly strong in our data—the effect was small (β=0.07 (CIT) versus β =-0.12*

(AD)) and became non-significant when examined in the perceptual domain only (S6 File)."

Q.1.27: Line 355: This is the first occurrence of "tutorial accuracy titration," which is not explained. These analyses should not be discussed if they are not justified and presented in the methods or supplementary materials.

R.1.27: We apologise for the lack of clarity in this instance. Participants completed 25 practice trials prior to each of the tasks, where the two-down one-up staircase procedure was already applied to minimize burn-in period. This was what we referred to as the tutorial accuracy titration. We note that we indicated this section under **Experimental Tasks** in the methods and have presented these results in the Supplementary (now **S1 File**) previously. For clarity. we now rephrased it to be more explicit:

"We considered several explanations for this through control analyses, and determined that it was not due to differences between the tasks in the (i) accuracy titrated during the tutorial…"

Q.1.28: Methods: The analyses are not sufficiently detailed. It is unclear which analysis was used to address which objective.

R.1.28: We apologise for the oversight of missing out including the global metacognition analyses, which have contributed to the lack of detail and confusion. We have now added this into the methods. Overall, there are three key sections of analyses (i) local confidence regressions with questionnaire/factor scores, (ii) global metacognition regressions with questionnaire/factor scores, and (iii) step-wise regressions and cross-validation. Parts (i) and (ii) are now detailed by the **General linear modelling** section and (iii) is under **Stepwise regression** and **Repeated cross validation**.

Q.1.29: What is the rationale for using meta-d' analyses? The use of metacognitive efficiency does not address any of the objectives of this study.

R.1.29: Meta-d' analyses were included due to two reasons. Firstly, there has been some suggestions of domain-specificity of metacognitive efficiency (see Palmer et al., 2014, Consciousness and Cognition), which we believed was worth exploring within our data. Secondly, prior work (Rouault et al., 2018) have found dissociable associations of anxious-depression and compulsivity, where higher anxious-depressive levels were associated with higher metacognitive efficiency, while higher compulsivity levels were associated with lower metacognitive efficiency. We did not replicate this effect, and thus we presented these analyses in the supplement (**SFile 3**).

Q.1.30: It is not clear if mixed models that account for both intra- and inter-subject variability were used. It seems that this might be the case, given certain R functions used (particularly lmer). However, the fitglm() function in Matlab was used for cross-validation, and this function does not fit mixed models. This suggests that different models were used for cross-validation. In any case, given repeated measures per subject, mixed models, including random effects for subjects, should be used. Also, regarding trial-by-trial confidence ratings, it is recommended to use mixed-effects models that include random effects for subjects, instead of averaging each subject's confidence ratings during the task and then including this average in a regression model.

R.1.30: We apologise for the confusion on these methods. To clarify, general linear models were used for the main analyses (lm() in R), and thus the cross-validation using fitglm() in Matlab fully replicates the same models. We included lmer() in the methods as mixed models were used to examine a supplemental analysis (**S2 File**), and this was a legacy mention. We have now removed this mention in the main text.

We used the mean of trial-by-trial confidence ratings as the local confidence measure for the simplicity of using a general linear model, and for similarity with the global metacognition analyses. We also note that prior work using these same methods have likewise used the average of local confidence ratings (Rouault et al., 2018, Benwell et al., 2022; Hoven et al., 2022, etc.) and that using trial-by-trial confidence ratings in a mixed-effect model with our current data showed the same results as the general linear model. For these reasons, we decided to keep the mean confidence measure in the analysis.

Q.1.31: Furthermore, it seems that only one model type (e.g., a regression model with Dimensions as the dependent variable and local + global metacognition + age + IQ + gender, and interactions with task domains as predictors) could have been used to discuss, all at once, local and global metacognitive contributions to anxious-depression and compulsivity.

R.1.31: We agree with reviewer that a regression including all the metacognitive metrics would be an alternative way to discuss the results than using the stepwise regression. However, we believe that the demonstration of the stepwise analyses has additional benefits of (i) determining the relative contribution of these metrics to the factor scores via model fit, given that the reviewer previously noted that the size of the regression coefficient was a qualitative interpretation and (ii) to elucidate the simplest explanatory model from a large number of potential regressors.

Q.1.32: Finally, regularized regression (e.g., LASSO or Ridge) would be more appropriate than stepwise regression, especially since out-of-sample predictions for compulsivity are poor and since multiple global metacognition measures are likely collinear.

R.1.32: We did consider regularised regression methods for the prediction of anxious-depression/compulsivity. However, we found that using an elastic net regression showed the same results as the stepwise. Key contributors for anxious-depression were the self-esteem (RSE), self-efficacy (GSE) and local confidence (task_conf) to anxious-depression, while it was self-esteem (RSE), self-efficacy (GSE), post-task confidence (task_postConf) and local confidence (task_conf) for compulsivity.

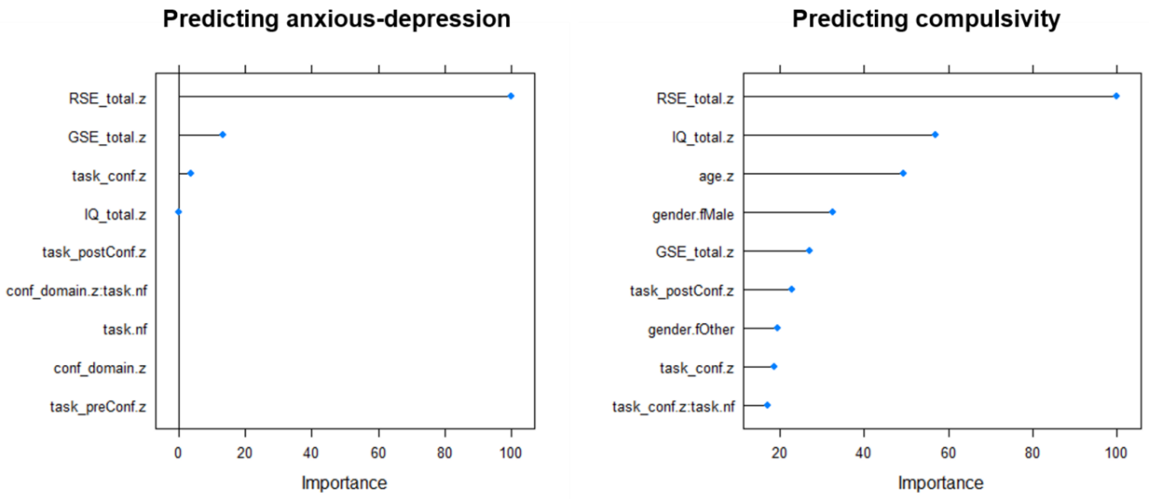

Ultimately, we felt that the stepwise approach presented a clearer and more straightforward demonstration of the contributions of metacognitive metrics to the factor scores.

Additionally, we also note that the global metacognitive measures, while positively correlated, were not particularly highly correlated (r=0.08-0.33) with the exceptions of pre- and post-task metacognition, and self-esteem / self-efficacy (**S7 File**).

Reviewer #2:

Q.2.1: This paper addresses an apparent dissociation in the empirical observations regarding metacognitive processes in the general population when confronted with psychopathological dimension or "symptoms" derived from self-questionnaires: while anxious-depression traits are associated with diminished confidence in one's cognitive performance in psychometric tasks, scoring high on compulsive and intrusive thoughts is associated with overconfidence in the same type of cognitive tasks. The study here proposed by Seow, Fleming and Hauser attempt to refine our understanding of such observations reported in previous papers of their groups and in those of other colleagues.

To do so, they contrast metacognitive performance metrics across two different tasks, a perceptual decision task vs. a working memory task, to generalize across domains. Testing those two tasks in the same participants (n=327) sampled from the general population thanks to online testing give the authors enough power to replicate earlier results across domains. It also sheds light on the aforementioned dissociation between the two psychopathological traits with respect to local (trial by trial) or global metacognitive assessments.

The strength of this report lays in the relatively large number of participants performing multiple well controlled tasks and responding to extensive questionnaires, all of which is analyzed through cogent statistical procedures. The authors deployed appropriate control procedure to ensure the quality of data collected online which is notoriously less reliable than that collected in the lab. The analyses are well-conducted. In general, the article is well constructed and easy to read.

R.2.1: We thank the reviewer for their positive comments.

Q.2.2: I will have very few remarks: Authors, as in earlier publications of theirs and others, describe self-report questionnaire scores as "symptoms" (e.g. "anxious depression symptoms"). However, in the general population it would be more appropriate to speak of subclinical traits or psychopathological dimensions. Especially given, the fact that, as they themselves note that observations in psychiatric patients (e.g. OCD) may not be entirely consistent with the outcome observed in association to traits like "compulsive impulsive". At least, the manuscript should explicitly acknowledge the slightly abusive use of the word "symptoms" when referring to psychological dimensions, defined in the general non-clinical population whose concurrent validity to actual psychopathological processes in patients remains to be demonstrated.

R.2.2: We agree with the raised point. We have now been more careful with the term 'symptoms', and have replaced every instance when it refers to questionnaire/factor scores of the general population sample:

> *"Second, different findings may arise due a mixture of psychopathological traits across the different samples [33]."*
>
> *"Thereafter, participants completed a battery of self-report questionnaires assessing a range of psychopathological subclinical traits…"*
>
> *"In this study, we sought to reconcile diverging findings in how metacognition relates to psychiatric symptoms/subclinical traits…"*
>
> *"We considered three possible reasons for this divergence: (i) a metacognitive domain-specificity leading to distinct results with memory and perception tasks, (ii) the co-occurrence of mental health traits overshadowing effects…"*
